# Use of BOIvy Optimization Algorithm-Based Machine Learning Models in Predicting the Compressive Strength of Bentonite Plastic Concrete

**DOI:** 10.3390/ma18133123

**Published:** 2025-07-01

**Authors:** Shuai Huang, Chuanqi Li, Jian Zhou, Xiancheng Mei, Jiamin Zhang

**Affiliations:** 1 School of Resources and Safety Engineering, Central South University, Changsha 410083, China; 2 State Key Laboratory of Geomechanics and Geotechnical Engineering, Institute of Rock and Soil Mechanics, Chinese Academy of Sciences, Wuhan 430071, China; xcmei@whrsm.ac.cn; 3 State Key Laboratory of Deep Geothermal Resources, SINOPEC Research Institute of Petroleum Engineering, Beijing 100101, China

**Keywords:** compressive strength, bentonite plastic concrete, Ivy algorithm, Bayesian optimization, model interpretability

## Abstract

The combination of bentonite and conventional plastic concrete is an effective method for projecting structures and adsorbing heavy metals. Determining the compressive strength (CS) is a crucial step in the design of bentonite plastic concrete (BPC). Traditional experimental analyses are resource-intensive, time-consuming, and prone to high uncertainties. To address these challenges, several machine learning (ML) models, including support vector regression (SVR), artificial neural network (ANN), and random forest (RF), are generated to forecast the CS of BPC materials. To improve the prediction accuracy, a meta-heuristic optimization, called the Ivy algorithm, is integrated with Bayesian optimization (BOIvy) to optimize the ML models. Several statistical indices, including the coefficient of determination (R^2^), root mean square error (RMSE), prediction accuracy (U_1_), prediction quality (U_2_), and variance accounted for (VAF), are adopted to evaluate the predictive performance of all models. Additionally, Shapley additive explanation (SHAP) and sensitivity analysis are conducted to enhance model interpretability. The results indicate that the best model is the BOIvy-ANN model, which achieves the optimal indices during the testing. Moreover, water, curing time, and cement are found to be more influential on the prediction of the CS of BPC than other features. This paper provides a strong example of applying artificial intelligence (AI) techniques to estimate the performance of BPC materials.

## 1. Introduction

Plastic concrete is widely used to protect critical engineering structures (such as earthen dams) because of its unique deformation characteristics and permeability [1]. As a novel construction material, plastic concrete consists of water, bentonite clay, cement, and aggregate, mixed in specific proportions to achieve high ductility and low permeability, offering advantages over traditional materials [2,3,4]. Among these components, Bentonite is a natural clay predominantly composed of montmorillonite, which confers its characteristic high swelling capacity. Therefore, bentonite plays a crucial role in enhancing the sealing properties of the material and structure [5]. Additionally, bentonite has been extensively applied in the adsorption of heavy metals [6]. However, while the inclusion of bentonite improves certain beneficial properties of bentonite plastic concrete (BPC), it also has an inevitable impact on key properties, such as compressive strength (CS) [7]. Therefore, accurately evaluating the strength performance of BPC materials is essential to ensure the safety and integrity of engineering or building structures.

One of the most direct and effective methods to observe changes in the properties of BPC and explore the effects of each component on CS is through laboratory experiments [8]. Kazemian et al. [9] investigated the effects of bentonite type, cement content, and mixing ratio of components on the CS and other performance characteristics of BPC materials. Their study showed that an increase in water content directly resulted in a decrease in BPC strength. Additionally, the influence of cement on BPC strength is found to be significant and cannot be ignored. Iravanian and Bilsel [10] conducted compression and “double punch” tests to assess the impact of curing time on both the CS and tensile strength (TS) of BPC materials. The results indicated that both CS and TS increased with longer curing time, particularly for cement-based materials. However, the CS increase of BPC was found to be lower than that of ordinary concrete under the same curing time [11]. Kazemian and Ghareh [12] examined the effects of gravel, sand, cement, water, and bentonite on BPC performance, including CS, elastic modulus, and permeability. The results demonstrated a direct positive correlation between the term activity ratio in bentonite and the CS of BPC materials. Furthermore, cement was shown to directly influence both the elastic modulus and permeability of BPC. Haq et al. [13] used bentonite or silica fume as partial replacements for cement in concrete materials and conducted tests on their mechanical properties. The results showed that the addition of bentonite significantly improved the compressive performance and durability of concrete. Tang et al. [14] performed uniaxial and triaxial tests on plastic concrete to measure its mechanical properties. Their research indicated that concrete strength was higher when the cement content was high and the bentonite content was low. Iravanian and Bilsel [15] discussed the influence of cement content on the strength characteristics of compacted sand–bentonite concrete. Their findings revealed that the CS and TS of the material increased by 300% with the addition of cement. Basha and Mansour [16] prepared 105 concrete samples to carry out performance tests. Their results indicated that the 7-day strength of concrete containing bentonite decreased by 35, which was 51% higher than that of the concrete without bentonite. While laboratory tests have demonstrated the effects of cement and bentonite content on the properties of BPC materials, these experiments are time-consuming and labor-intensive, making the optimization of concrete material design challenging [17,18]. Therefore, a new approach is needed to integrate the analysis of influencing factors with the prediction of BPC performance.

With the rapid developments in computer science driven by software upgrades and hardware iterations, artificial intelligence (AI) technologies represented by machine learning (ML) have become widely applied across various fields, including structural engineering, materials science, civil engineering, water conservancy, and mine engineering [19,20,21,22,23,24,25]. In the context of predicting the performance of BPC, Inqiad et al. [26] employed multi-expression programming (MEP), AdaBoost, and gene expression programming (GEP) to predict the 28-day CS of BPC materials. Their results revealed that the AdaBoost model achieved the optimal prediction accuracy, with a root mean square error (RMSE) of 1.66. Ghanizadeh et al. [27] developed an artificial neural network (ANN) and a support vector regression (SVR) model using 72 concrete samples to predict the CS of BPC. They found that both intelligent models demonstrated satisfactory prediction accuracy, with sand content identified as the most significant feature for predicting CS through sensitivity analysis. Khan et al. [28] reported an example of the application of an MEP model to CS prediction. The results showed that the MEP outperformed traditional regression methods in prediction accuracy, with water content identified as the most influential parameter. Alishvandi et al. [29] used 645 samples with six features to develop six ML models (e.g., random forest (RF) and SVR) for predicting the CS of plastic concrete. Their results indicated that the RF model outperformed the other five models and provided insights into the contribution of different features to CS prediction. To further improve the prediction accuracy, meta-heuristic optimization algorithms were utilized to select the optimal hyperparameters for the ML models. Kumar et al. [30] developed several extreme gradient boost (XGBoost) models to predict the CS of BPC materials. These models were optimized by genetic (GA), particle swarm optimization (PSO), and dragonfly optimization (DO) algorithms. The prediction results demonstrated that the optimal model (PSO-XGBoost) obtained a high predictive performance, with a high value (0.974) for the coefficient of determination (R^2^). Thapa et al. [7] integrated grey wolf optimization (GWO), the firefly algorithm (FA), GA, PSO, gradient-based optimization (GBO), and cultural algorithms (CAs) with ANN models to predict the CS of BPC materials. Among all hybrid models, the FA-ANN model obtained the highest prediction accuracy of 96% among all hybrid models. However, some newer meta-heuristic optimization algorithms (such as the Ivy algorithm) with obvious performance advantages have not yet been applied to CS prediction in combination with ML models. Among ML models, the ANN model excels at capturing the potential relationship between input features and output features [31], the SVR model is particularly effective for small sample sizes with high prediction accuracy [32], and the RF model has a strong advantage in preventing model overfitting [33]. Additionally, attention must be paid to the issue of population initialization based on the random strategy that easily causes the hybrid model to fall into a local optimum. Bayesian optimization (BO) offers advantages in enhancing optimization performance and computational efficiency [34]. For population initialization, BO can estimate the objective function by constructing a probabilistic model, thereby avoiding extensive random sampling. Moreover, BO is capable of identifying potentially promising search regions at the early stage of initialization, thus reducing computational resource waste [35]. Studies such as Soares et al. [36] have further improved the prediction accuracy of PSO-ANN models by integrating BO. However, the application of BO in combination with several meta-heuristic optimization algorithms for predicting the CS of BPC materials has not yet been explored.

Therefore, this paper aims to develop SVR, ANN, and RF models to predict the CS of BPC materials. A comprehensive database consisting of 169 samples established by Amlashi et al. [37] is adopted to train the model proposed in this work. Furthermore, a meta-heuristic optimization algorithm, called the Ivy algorithm, is utilized to optimize the three prediction models. To avoid the model falling into a local optimum, the BO algorithm is further combined with the Ivy algorithm to achieve the optimal selection of hyperparameters. The remainder of this paper is organized as follows: Section 2 introduces the principles of the ML models and optimization algorithms adopted in this paper; Section 3 describes the samples used for training the models; Section 4 outlines the entire process of developing the prediction models and evaluating their performance; Section 5 presents the results of discussions on CS prediction; and the main conclusions are summarized in Section 6.

## 2. Methodologies

### 2.1. SVR

**Figure 1:**
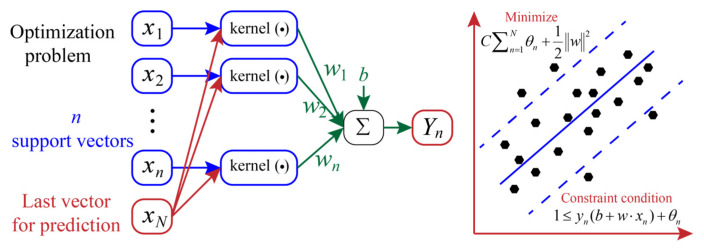
The structure of the SVR model used for solving the regression problem.

SVR is a variant of the support vector machine (SVM) model, specifically designed to solve regression problems [38]. Unlike traditional regression methods that rely on fitting data points, SVR constructs a regression model that allows for certain errors to achieve the best prediction. As shown in Figure 1, SVR fits data points as closely as possible by constructing a hyperplane while minimizing model complexity. The model complexity is determined by the penalty parameter (*C*). Additionally, kernel functions represented by radial basis function (RBF) are commonly used to improve model generalization, i.e., mapping data to higher-dimensional feature spaces, so complex regression problems can be solved by building linear hyperplanes in this space [39]. Thus, for a regression problem, the minimum error in SVR can be obtained by using the following formula:

(1) $$\begin{array}{l} \mathrm{Minimize}:C\sum_{n=1}^{N}\theta_{n}+\frac{1}{2}{\left\|w\right\|}^{2}\\ \mathrm{Constraint}\;\mathrm{condition}:1\leq y_{n}(b+w\cdot x_{n})+\theta_{n} \end{array}$$

where *N* represents the number of data points. $\theta_{n}$ represents the distance between the *n*-th data point and its margin hyperplane. *w* and *b* are the normal vector and bias of the hyperplane, respectively. $x_{n}$ and $y_{n}$ belong to the dataset consisting of *N* samples. In this paper, the RBF with a key inner parameter (*g*) was combined with the SVR model to minimize the prediction errors.

### 2.2. ANN

**Figure 2:**
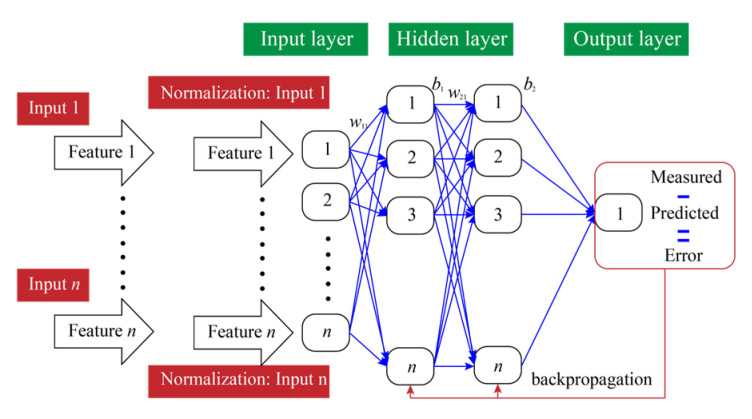
The structure of the ANN model used for solving the regression problem.

ANN is an ML model that simulates the structure of human brain neurons and is commonly used to solve nonlinear regression tasks [40]. As illustrated in Figure 2, the basic structure of an ANN model consists of an input layer, one or more hidden layers (*N_h_*), and an output layer, with each layer comprising several neurons (*N_n_*). Input features are passed into activation functions through weighted summation, and nonlinearity is introduced to enhance the model’s fitting ability. During the training process, the backpropagation (BP) algorithm is typically used to minimize the loss function, iteratively adjusting the weights and thresholds to improve the prediction accuracy. The value of each neuron can be calculated using Equation (2). The key advantage of an ANN model is its ability to automatically learn the complex mapping relationship between input and output, enabling efficient modeling and prediction of regression targets [41].

(2) $$V_{i}=f\left(\sum_{i=1}^{I}w_{i}a_{i}-b_{i}\right)$$

where $V_{i}$ represents the value of the *i*-th neuron in the current layer. $w_{i}$ and $b_{i}$ represent the weights of the *i*-th neuron between the adjacent layers and bias values in the current layer, respectively. $a_{i}$ is the value of the *i*-th neuron in the previous layer. *I* is the maximum number of neurons in the current layer.

### 2.3. RF

**Figure 3:**
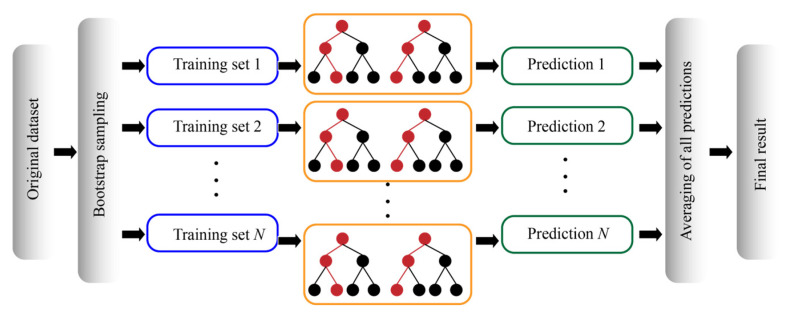
The structure of the RF model used for solving the regression problem.

RF is an ensemble learning method that improves the stability and accuracy of a model by constructing multiple decision trees (DTs) and averaging their prediction results (see Figure 3). During model training, samples were selected from the database to establish a DT according to the sampling with replacement (i.e., the Bootstrap rule), and features were randomly selected at each node split, which increases model diversity and reduces the overfitting risk. For the RF model, the selection of the number of trees (*N_t_*) and minimum leaf point (*Minleafsize*) significantly impacts the model’s prediction accuracy. The former is closely related to model stability and computational efficiency, while the latter influences the data fitting process. In general, RF models are widely used for regression problems due to their strong performance in handling nonlinear relationships and feature redundancy [42].

### 2.4. Ivy Algorithm with Bayesian Optimization

The Ivy algorithm was developed by Ghasemi et al. [43] to solve various optimization problems, inspired by simulating the life stages (e.g., growing, rising, and spreading) of ivy plants. The algorithm establishes mathematical and experimental models based on the growth rate of ivy plants, determines the growth direction using ivy plant knowledge, and mimics the plant’s behavior through a self-improvement mechanism. Optimization tasks using this algorithm generally follow the following steps:

(1) Population initialization

Assuming that each ivy plant is a candidate solution to an optimization problem, the initial position of each ivy plant is determined according to a random distribution strategy in a certain search space. This principle can be expressed mathematically as follows:

(3) $$PI_{i}=I_{lb}+rand\cdot (I_{ub}-I_{lb})$$

where $PI_{i}$ represents the initial position of the *i*-th ivy plant. $I_{ub}$ and $I_{lb}$ represent the upper and lower boundaries of the searching space, respectively. *rand* () is a random number within the range of 0 to 1.

(2) Population growth

As a trailing plant that grows over time, the growth rate of the ivy plant is calculated as follows:

(4) $$\frac{dG_{v}(t)}{dt}=\psi \cdot G_{v}(t)\cdot \varphi (G_{v}(t))$$

where $G_{v}(t)$ and $\varphi (G_{v}(t)$ represent the growth rate and velocity of the ivy plant at the *t*-th iteration, respectively. ψ represents a correction factor.

(3) Growth with sunlight

**Figure 4:**
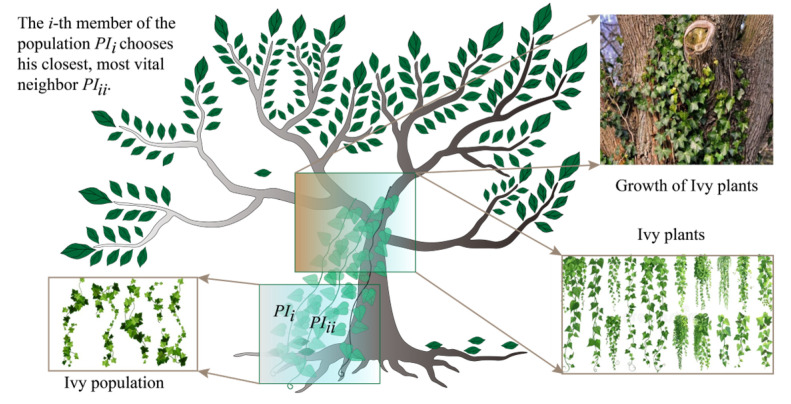
Illustration of the Ivy algorithm used for solving optimization problems.

The growth of ivy plants is closely tied to the availability of light sources, and observations in nature show that young ivy continues to grow on older ones, with the latter depending on the former’s growth for survival. This survival strategy is modeled in the algorithm through a neighbor selection and self-improvement mechanism (see Figure 4). Therefore, the new position of an ivy plant can be determined as follows:

(5) $$PI_{i}^{new}=PI_{i}+v\cdot \left(PI_{ii}-PI_{i}\right)+v\cdot \Delta G_{vi}$$

where $PI_{i}^{new}$ represents the new position of the *i*-th ivy plant in the current iteration. $PI_{ii}$ represents the position of the *i*-th ivy plant’s neighbor. *v* is a vector related to the direction of thr light source.

(4) Spreading and evolution

After finding the most important neighbor, the other ivy tries to get closer to the ivy in the best position for searching for better candidate solutions nearby:

(6) $$PI_{i}^{new}=PI_{best}\cdot \left(rand+v\cdot \Delta G_{vi}\right)$$

where $PI_{best}$ represents the best position of the plant in the current iteration.

For the BO algorithm, the core idea is to estimate the objective function and constraints through an agent model [44]. On this basis, an acquisition function is used to identify candidate solutions that are worth further evaluation. To enhance the optimization performance of the Ivy algorithm and avoid entrapment in local optima during hyperparameter tuning, BO is integrated into the population initialization stage. The process follows several key steps. (1) Surrogate model construction: A Gaussian process (GP) is chosen as an agent model to handle both inequality and equality constraints, and a collection function based on the expected improvement is chosen to generate candidate solutions [45,46]. This model not only estimates the function values but also quantifies uncertainty. (2) Acquisition function definition: An acquisition function, expected improvement (EI), is defined to balance exploration and exploitation by favoring points that either have high predicted values or high uncertainty. (3) Candidate sampling: The acquisition function is maximized to select the most promising hyperparameter configurations. (4) Evaluation and update: The selected candidate is evaluated using the true objective function, and the resulting data are used to update the surrogate model. (5) Population initialization: The top-performing candidates identified through BO are used to initialize the population of the Ivy algorithm, ensuring a more informed and diverse starting point. The pseudo-code is introduced in Algorithm 1. In this paper, BO is used to establish a fitting relationship between the input features and the target values based on the basis of Ivy’s optimization. The corresponding Gaussian regression formula can be expressed as:

(7) $$f\left(x\right)∼GP(m\left(x\right),k(x,x^{\prime }))∼GP\left(0,\sigma_{f}^{2}\exp(-\frac{1}{2l^{2}}{\left\|x-x^{\prime }\right\|}^{2}\right)$$

where $m\left(x\right)$ and $k(x,x^{\prime })$ represent the mean function and covariance function, respectively. In this paper, the mean function was set to 0 and the radial basis function (RBF) kernel was considered as the covariance function. $\sigma_{f}^{2}$ and l need to be set manually. After that, the candidate needs to be further evaluated using Equation (8).

(8) $$EI(x^{*})=E[\max\left(f\left(x^{*}\right)-y_{\min},0\right)]=\sigma \left(x^{*}\right)\left(\frac{\mu \left(x^{*}\right)-y_{\min}}{\sigma \left(x^{*}\right)}\Phi (\frac{\mu \left(x^{*}\right)-y_{\min}}{\sigma \left(x^{*}\right)})+\phi (\frac{\mu \left(x^{*}\right)-y_{\min}}{\sigma \left(x^{*}\right)})\right)$$

where Φ(·) and ϕ(·) represent the cumulative distribution function and the probability density function of the standard normal distribution, respectively.
**Algorithm 1** Pseudo-code of BO-enhanced Ivy initialization.Input
Initialize dataset *D* = {}
for *t* in range (1, T + 1):
if *t* == 1:
x_t = RandomSample ()
else:
Fit GP model on *D*
Define acquisition function (expected improvement)
x_t = argmax_x AcquisitionFunction(x)
y_t = EvaluateObjectiveFunction (x_t)
*D* = D ∪ {(x_t, y_t)}
TopCandidates = SelectTopK (*D*, k)
InitializeIvyPopulation (TopCandidates)
RunIvyOptimization()
End

## 3. Database

**Figure 5:**
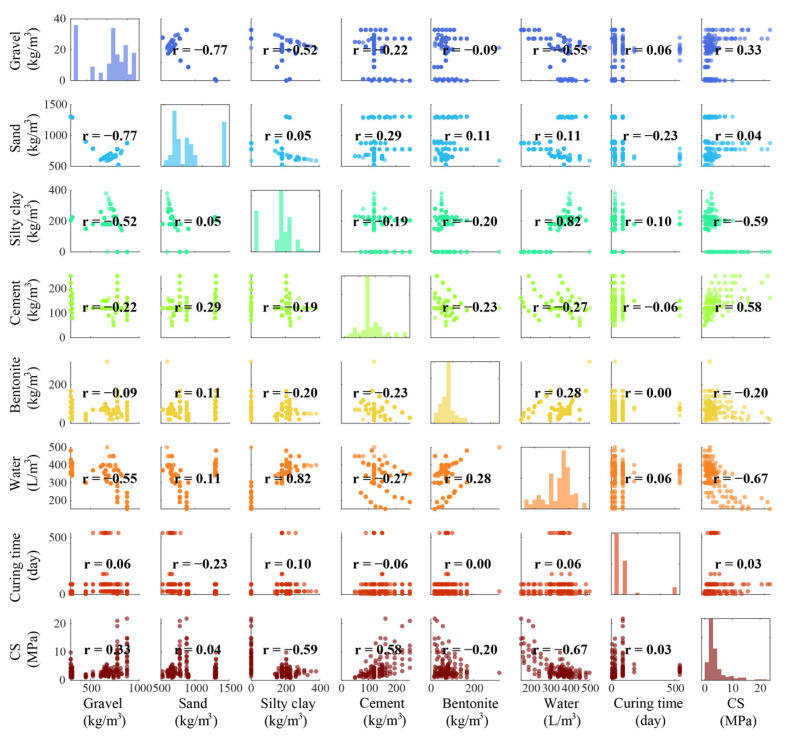
Illustration of the Ivy algorithm used for solving optimization problems.

As mentioned in Section 1, several parameters (such as gravel, sand, cement, water, and bentonite) have a significant effect on BPC’s performance, especially its CS. To construct a high-performance model and determine the influence of these parameters on the CS of BPC materials, a dataset consisting of 169 samples developed by Amlashi et al. [37] was utilized to generate prediction models. This dataset includes seven parameters, i.e., gravel, sand, silty clay, cement, water, bentonite, water, and curing time, which were considered as input features to train the models for predicting CS. The statistical results of these features are illustrated in Table 1. Furthermore, the correlation between features needs to be calculated to remove features that negatively affect model performance [47]. Therefore, the Pearson correlation coefficient method was used to calculate the linear correlations between the features (see Figure 5). As shown in this picture, the highest correlation coefficient between input features is −0.77, and the lowest value is zero. These results suggest that the influence of each input feature on the prediction target (i.e., CS) is independent. Moreover, the correlation coefficients between silty clay (−0.59) and cement (0.58) and the CS are significantly higher than those between the other features and the CS. In general, all selected features are considered relevant for the subsequent model development.

## 4. Development of Prediction Models

**Figure 6:**
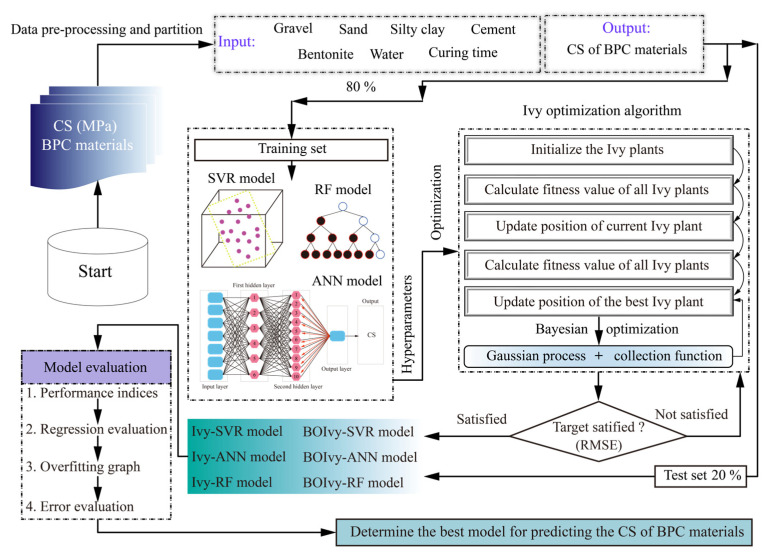
The framework of predicting BPC’s CS based on the developed models.

In this paper, three ML models (SVR, ANN, and RF) were developed using 169 samples to predict the CS of BPC materials. To further improve the prediction accuracy of models, the initial Ivy algorithm and its variant optimized by BO were utilized to select the best combinations of hyperparameters for all models. As illustrated in Figure 6, the whole process of developing the prediction models can be divided into the following steps:

(1) Data preparation

As mentioned earlier, the dataset consists of 169 samples used to generate the prediction models. According to the data allocation principle, the majority of the data are allocated to the training set to ensure the model has sufficient information to learn the relationship between input features and the output feature. The remaining data are allocated to the test set to validate the performance of the trained models. Therefore, the data allocation ratio is set at 8:2 in this paper, i.e., the training set includes 135 samples, and the remaining 34 samples are packaged into the test set. Additionally, all data need to be normalized into the range of [0, 1] or [−1, 1] to prevent dimensional differences from weakening model performance.

(2) Hyperparameter optimization

After determining the ratio of training sets to test sets, SVR, ANN, and RF models were generated to predict the CS of BPC materials. However, the performance of these models is largely dependent on the choice of hyperparameters. For the SVR models, *C* and *g* were set within the range of [0.25–128] and [0.25–16], respectively. For the ANN model, *N_h_* and *N_n_* were set within the range of [1, 2] and [1–10], respectively. For the RF model, *N_t_* and *Minleafsize* were set within the range of [1–100] and [1–10], respectively. Additionally, the optimization performance of both algorithms is also controlled by several parameters, including population size and the number of iterations [48]. When the population size is small, the model may fall into local optimality, while the large number of iterations will lead to a waste of computing resources. Accordingly, population sizes of 25, 50, 75, and 100 were tested for selecting the optimal hyperparameters of all models during 200 iterations (see Table 2). Furthermore, a fitness function must be determined to evaluate the candidate solutions during the iterations. Several statistical indices, including mean square error (MSE), RMSE, and R^2^, are typically used to define fitness functions [49]. Among these, RMSE is widely chosen to calculate the fitness values because it does not take absolute values into account [50]. In addition, a five-fold cross-validation was also adopted to further improve optimization ability and to avoid overfitting. The fitness function can be defined as follows:

(9) $$fitness=\frac{1}{K}\sum_{k=1}^{K}RMSE_{k}$$

where *K* represents the maximum number of subsets in the cross validation.

(3) Model evaluation

After obtaining the optimal parameters for all models, the test set was utilized to verify the model performance and determine the best model for predicting the CS of BPC materials. Some classical statistical indices were utilized to compare the prediction performance between all models, including R^2^, RMSE, prediction accuracy (U_1_), prediction quality (U_2_), and variance accounted for (VAF) [51,52,53]. Among them, R^2^ indicates how well the model explains the variance in the actual output. A value closer to 1 suggests a better fit between the model and the observed data. RMSE measures the average deviation between the predicted and actual values, where a smaller value implies higher prediction accuracy. U_1_ evaluates the proportion of prediction error relative to the overall magnitude of both actual and predicted values. A lower U_1_ value reflects greater predictive precision. U_2_ represents the proportion of the prediction error in relation to the total variation in the actual values. Smaller U_2_ values indicate better prediction quality. VAF measures the extent to which the model explains the variance in the actual values, expressed as a percentage. A value closer to 100 percent means the model more effectively captures the trend of the data variation. These indices are defined using Equations (10)–(14). In addition, various evaluation tools were used to assist in model selection, such as regression graphs, overfitting graphs, and error analysis.

(10) $$R^{2}=1-\frac{{\left[\sum_{i=1}^{n}\left(a_{i}-p_{i}\right)\right]}^{2}}{{\left[\sum_{i=1}^{n}\left(a_{i}-\bar{a}\right)\right]}^{2}}$$

(11) $$\mathrm{RMSE}=\sqrt{\frac{1}{n}\sum_{i=1}^{n}{\left(a_{i}-p_{i}\right)}^{2}}$$

(12) $$U_{1}=\frac{\mathrm{RMSE}}{\sqrt{\frac{1}{n}\sum_{i=1}^{n}a^{2}}+\sqrt{\frac{1}{n}\sum_{i=1}^{n}p^{2}}}$$

(13) $$U_{2}=\frac{{\sum_{i=1}^{n}\left(p_{i}-a_{i}\right)}^{2}}{{\sum_{i=1}^{n}\left(a_{i}\right)}^{2}}$$

(14) $$\mathrm{VAF}=\left[1-\frac{\mathrm{var}(a_{i}-p_{i})}{\mathrm{var}(a_{i})}\right]\times 100\%$$

where $a_{i}$ and $p_{i}$ represent the actual and predicted values of the *i*-th data point, respectively. $\bar{a}$ represents the average value of the actual CS values in this paper. *n* is the maximum number of data points.

## 5. Results and Discussion

### 5.1. Model Optimization

**Figure 7:**
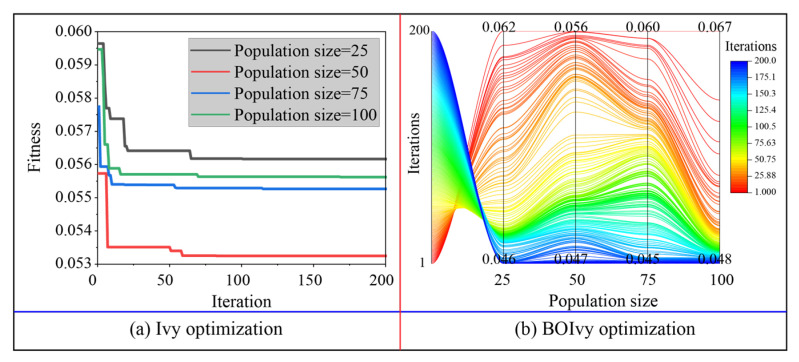
The iteration processes of SVR models optimized by the Ivy and BOIvy algorithms.

**Figure 8:**
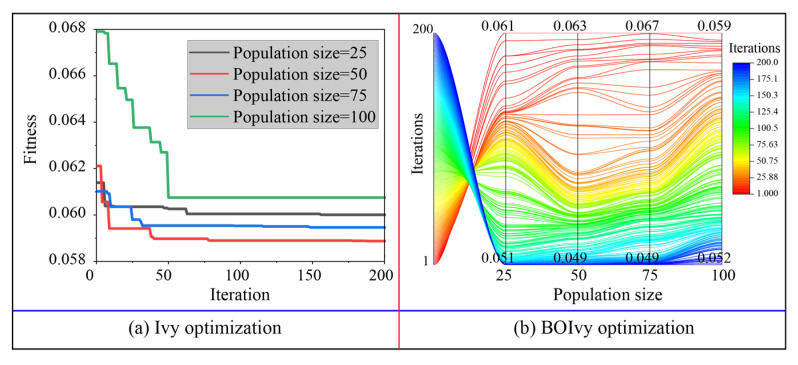
The iteration processes of ANN models optimized by the Ivy and BOIvy algorithms.

**Figure 9:**
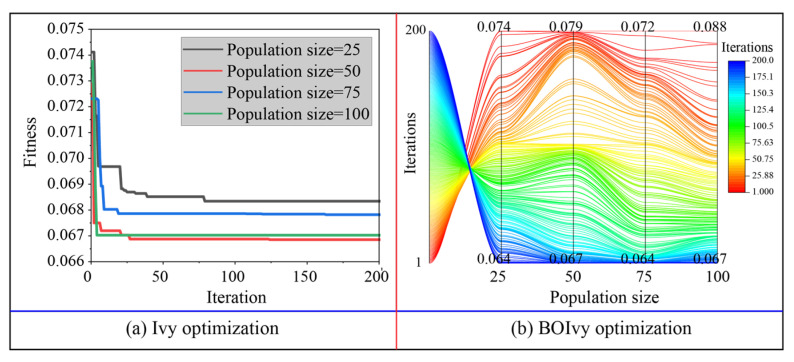
The iteration processes of RF models optimized by the Ivy and BOIvy algorithms.

The optimization results for the three models predicting the CS of BPC materials based on the training set are demonstrated in Figure 7, Figure 8 and Figure 9. For the SVR models, the minimum fitness values of all models optimized by Ivy with different population sizes were obtained before reaching the maximum number of iterations. Among these hybrid Ivy-SVR models, the model optimized by the Ivy algorithm with 50 ivy plants obtained the lowest fitness value during 200 iterations (see Figure 7a). On the other hand, the fitness values of all SVR models decreased when using the BOIvy algorithm to select the optimal combinations of hyperparameters. As shown in Figure 7b, the model optimized by the BOIvy algorithm with 75 ivy plants achieved the lowest fitness value after 200 iterations. Furthermore, the iteration curves of the ANN models optimized using the Ivy and BOIvy algorithms are illustrated in Figure 8. It can be observed that the Ivy-ANN model with a population size of 50 resulted in a lower fitness value compared to other models with population sizes of 25, 75, and 100. Similarly, the fitness values of ANN models optimized by the BOIvy algorithm were lower than those of the Ivy-ANN models. Additionally, Figure 9 shows the iteration curves of RF models optimized using both the Ivy and BOIvy algorithms. It can be seen that the minimum fitness values of the Ivy-RF or BOIvy-RF models with population sizes of 50 and 100 are close to and lower than those of the other models. As demonstrated in Table 3 and Table 4, when the population size is fixed, the SVR models consistently show lower fitness values than the other two types of models, especially the RF models. Overall, the optimization results indicate that using BO to improve the initial Ivy algorithm is beneficial for finding the best hyperparameter combinations for each model. As a result, the optimal hyperparameters of the SVR, ANN, and RF models determined by the Ivy and BOIvy algorithms are listed in Table 5.

### 5.2. Model Performance Evaluation

After determining the optimal hyperparameters for each model, the performance of the models was compared to select the best one for predicting the CS of BPC materials. The results for the evaluation indices of all hybrid models calculated using the training set are demonstrated in Table 6. It can be observed that the models optimized by the BOIvy algorithm delivered more satisfactory performance than those optimized by initial Ivy algorithms, achieving higher values of R^2^ and VAF, as well as lower values of RMSE, U_1_, and U_2_. Among these models, the BOIvy-SVR model obtained the best predictive performance, with an R^2^ of 0.9949, RMSE of 0.2267, U_1_ of 0.0115, U_2_ of 0.0021, and VAF of 99.4866%. However, the Ivy-RF model showed the poorest predictive performance, with an R^2^ of 0.9482, RMSE of 0.7194, U_1_ of 0.0738, U_2_ of 0.0443, and VAF of 94.8176%.

**Figure 10:**
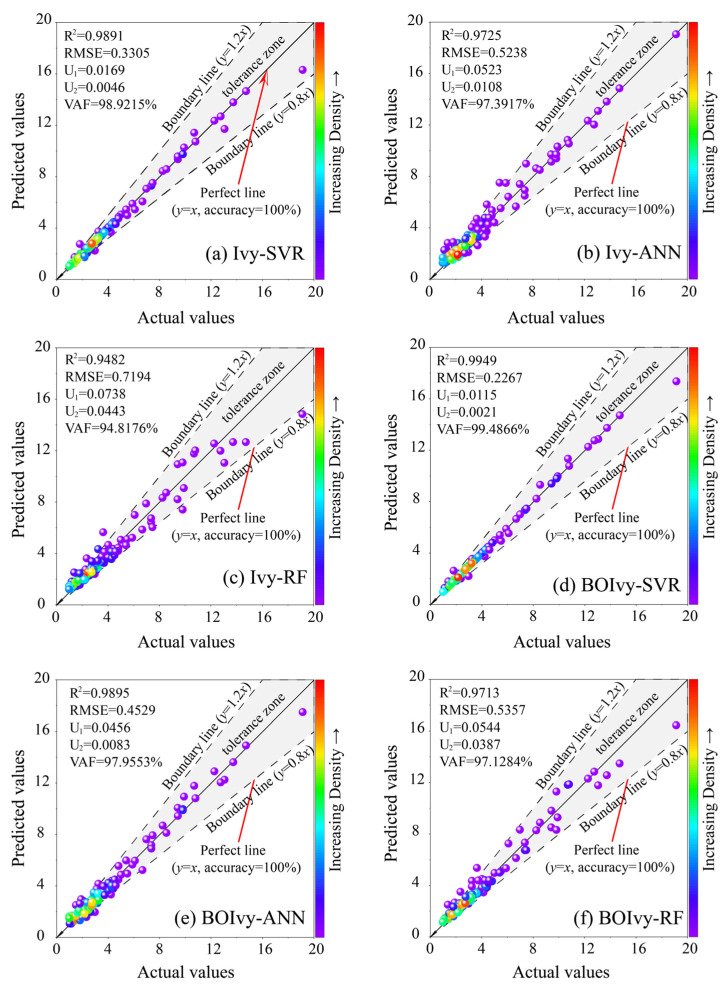
Regression graphs for all models optimized by the Ivy and BOIvy algorithms in the training phase.

Furthermore, a regression graph was generated to evaluate model performance by comparing the difference between the actual and predicted CS values. In this graph, the position of each data point is determined by the actual and predicted CS values. If the actual value matches the predicted CS value, the data point lies on the diagonal (*y* = *x*). Otherwise, the data points are distributed around the diagonal according to the degree of difference. Since most predictions inevitably involve some degree of error, this study introduces two boundary lines (*y* = 1.2*x* and *y* = 0.8*x*) to define an acceptable error region, referred to as the tolerance zone. Data points falling far outside this region are considered to result from low-quality predictions. Therefore, the number of points located within the tolerance zone and closer to the diagonal line serves as a key indicator for evaluating the model’s predictive performance. As shown in Figure 10a–c, most of the data points obtained by the Ivy-SVR model are located near the diagonal line and its adjacent areas. Some data points obtained by the Ivy-ANN model are farther from the diagonal line but still within the tolerance zone. However, most data points from the Ivy-RF model are scattered, with some values even exceeding the tolerance zone. The regression graph and the performance evaluation results of Ivy-based models are highly similar to those obtained by BOIvy-based models (see Figure 10d–f).

**Figure 11:**
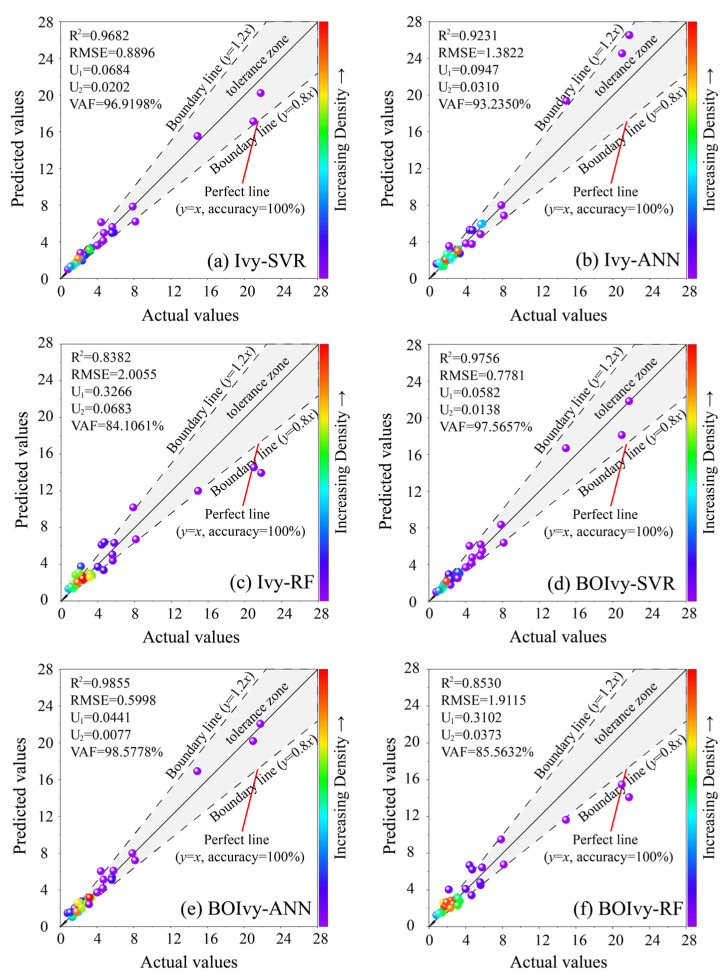
Regression graph for all models optimized by the Ivy and BOIvy algorithms in the testing phase.

On the other hand, a reliable model needs to not only perform well on the training set but also be validated by the test set. Table 7 presents the evaluation indices of all hybrid models calculated using the test set. It can be observed that all models exhibit a significant discrepancy between their performance indices on the test set and those on the training set, except for the BOIvy-ANN model. This model achieved the most satisfactory performance in the testing phase, with the highest values of R^2^ and VAF (0.9855 and 98.5778%) and lowest values of RMSE, U_1_, and U_2_ (0.5998, 0.0441, and 0.0077). The worst model in the testing phase is also the Ivy-RF model, with R^2^ of 0.8382, RMSE of 2.0055, U_1_ of 0.3266, U_2_ of 0.0683, and VAF of 84.1061%. The regression graphs of all models optimized by the Ivy and BOIvy algorithms in the testing phase are demonstrated in Figure 11. Compared to the RF models optimized by the Ivy and BOIvy algorithms, the data points for both Ivy and BOIvy-based models (i.e., Ivy-SVR, Ivy-ANN, BOIvy-SVR, and BOIvy-ANN) are more closely distributed along the diagonal or within the tolerance zone. These results indicate that the predictive performance of RF models is lower than that of the other models.

**Figure 12:**
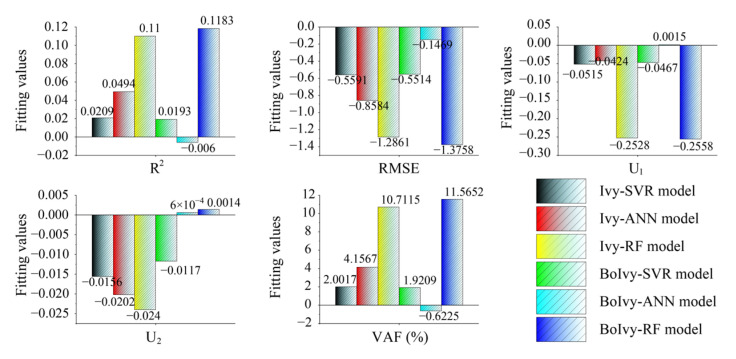
Demonstration of overfitting for all developed models.

Additionally, a fitting evaluation graph was constructed to show the performance difference between the model on the training set and the test set. Typically, the error between the evaluation indices of the model on the training set and the test set serves as the fitting evaluation index. Assuming that the model performs well on the training set, then an error equal to zero indicates that the model has neither underfit nor overfit and can be considered the best prediction model [54,55,56]. In this paper, five statistical indices, including R^2^, RMSE, U_1_, U_2_, and VAF, were used to generate five fitting evaluation graphs, as shown in Figure 12. It can be observed that the BOIvy-ANN model has the best performance in all fitting evaluations, i.e., the smallest errors in all five evaluation indices (R^2^: −0.006, RMSE: −0.1469, U_1_: 0.0015, U_2_: 6 × 10^−4^, and VAF: −0.6225). In contrast, the Ivy-RF model exhibits significant overfitting compared to other models, with R^2^ of −0.11, RMSE of −1.2861, U_1_ of −0.2528, U_2_ of −0.024, and VAF of 10.7115.

**Figure 13:**
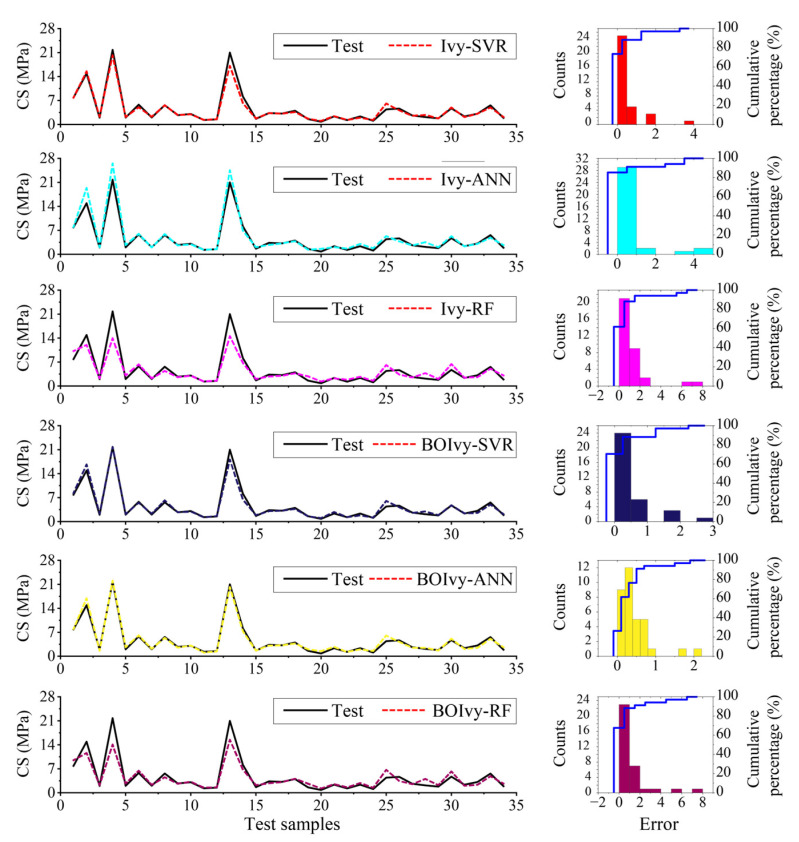
Comparison of curves for all developed models using the test set.

Next, error analysis was conducted to determine the best model for predicting the CS of BPC materials. Curves of the actual and predicted CS for all models are shown in Figure 13. It can be observed that the curves of the actual and predicted values align well in most areas, indicating that the predictive performance of the developed models is generally acceptable. However, most models showed high error predictions for the 4th, 14th, and 25th samples. On the other hand, for the BOIvy-ANN and BOIvy-SVR models, the curves of the predicted and actual values closely coincide, which suggests that their prediction performance is superior to that of the other models. The results of statistical error analysis are presented in Table 8. It can be observed that the Ivy-RF and BOIvy-RF models yielded the highest errors, with maximum errors exceeding 7 and the total errors surpassing 37. In contrast, the BOIvy-ANN model exhibited the smallest maximum error of only 2.0187, and its total errors were under 15. Therefore, the BOIvy-ANN was considered the optimal model for predicting the CS of BPC materials.

### 5.3. Model Interpretability

**Figure 14:**
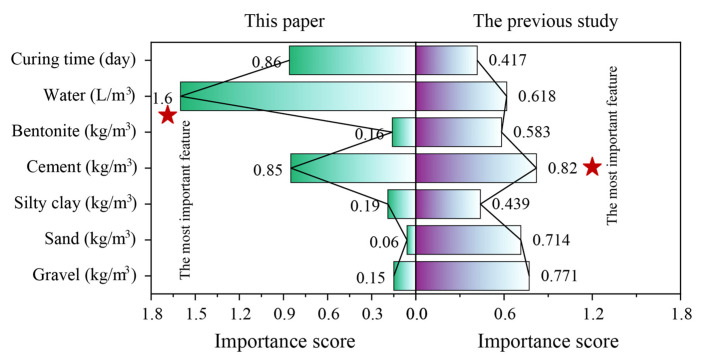
Demonstration of importance scores for each input feature based on the optimal model.

Although the optimal prediction model was determined, the influence of features on the CS prediction remained unclear. In other words, the model functioned as a “black box” and could not be transparently applied in practical projects. To address this, sensitivity analysis was carried out to calculate the importance score of each feature for predicting the target. Figure 14 demonstrates the results of the importance calculation for each feature based on the optimal model (the BOIvy-ANN model). It can be seen that water received the highest importance score (1.6) among all features. Following this, curing time and cement also had relatively high importance scores compared to the other features. However, in a previous study by Amlashi et al. [37], cement was identified as the most important feature for predicting the CS of BPC materials. The difference in conclusions between the two studies can be attributed to the variation in data sequences and the selection of hyperparameters used in modeling, which likely influenced the importance of each feature in predicting the same target.

**Figure 15:**
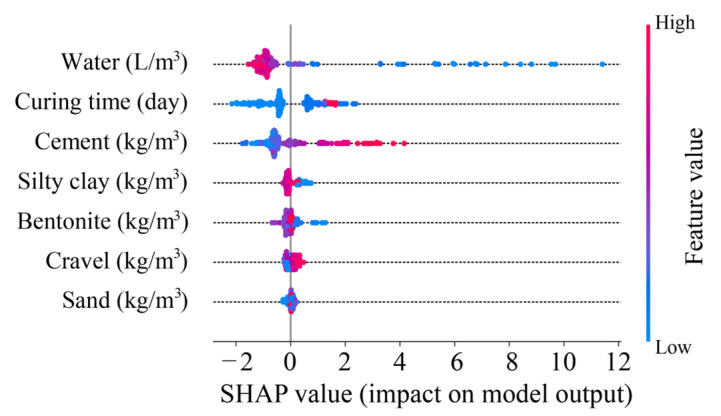
Contribution of features to CS prediction through SHAP analysis.

On the other hand, understanding the contribution of features to CS prediction is essential for optimizing the design of BPC materials. To achieve this, the Shapley additive explanation (SHAP) analysis was conducted. As shown in Figure 15, the points with colors from blue to red represent the values of features from low to high. When blue points are located in the positive SHAP value region, the corresponding feature exhibits a negative correlation with the predictive target. Conversely, features with red points in the positive SHAP value region indicate a positive correlation with the target. Based on the results of the SHAP analysis, water, silty clay, and bentonite provide negative contributions to CS prediction, and the other features are positively correlated with CS, especially curing time and cement.

## 6. Conclusions

In this research, a database consisting of 169 samples was utilized to train three ML models (SVR, ANN, and RF) for predicting the CS of BPC materials. The Ivy algorithm combined with BO was utilized to select the optimal hyperparameters of all models. Next, several statistical indices and tools were adopted to identify the best model. Finally, sensitivity and SAHP analyses were conducted to determine the importance score of features and their contributions to CS prediction. The main conclusions are summarized as follows:

(1) The results of model optimization indicated that BO significantly improved the optimization ability of the original Ivy algorithm, though the performance improvement for the RF model was relatively limited.
(2) The results of the model evaluation demonstrated that the BOIvy-ANN model outperformed the other models in predicting the CS of BPC materials, achieving the optimal indices with the test set (R^2^: 0.9855, RMSE: 0.5998, U_1_: 0.0441, U_2_: 0.0077, and VAF: 98.5778%) and the lowest fitting evaluation indices (R^2^: −0.006, RMSE: −0.1469, U_1_: 0.0015, U_2_: 6 × 10^−4^, and VAF: −0.6225)
(3) The results of the model explanation illustrated that water was the most important feature in predicting CS and had a negative contribution. Additionally, curing time and cement also played significant roles in CS prediction.

Although this study utilized 169 samples and employed the BOIvy algorithm to improve model performance, the relatively limited sample size may affect the generalizability and applicability of the models under different conditions. In addition, only a limited number of feature variables were considered, while other potential factors influencing the compressive strength of BPC materials, such as environmental temperature and types of admixtures, were not included. More samples need to be collected to enhance the robustness and applicability of the models by incorporating more diverse data. Moreover, additional relevant features should be introduced, and advanced machine learning algorithms or ensemble methods should be explored to further improve the accuracy and interpretability of CS predictions. Furthermore, the current study did not incorporate natural, climatic, or anthropogenic factors that may affect the long-term performance of BPC materials, which should be addressed in future research to improve real-world applicability.

## Figures and Tables

**Table 1:** Statistical results of input and output features.

Features	Unit	Statistical Indices			
		Mean	St. D	Max	Min
Gravel	kg/m^3^	616.46	191.41	875.00	295.00
Sand	kg/m^3^	840.05	255.94	1305.00	524.00
Silty clay	kg/m^3^	160.18	92.76	380.00	0.00
Cement	kg/m^3^	134.54	39.75	252.00	50.00
Bentonite	kg/m^3^	72.43	38.72	320.00	16.00
Water	L/m^3^	336.71	77.25	500.00	152.10
Curing time	day	83.54	131.79	540.00	7.00
CS	MPa	3.98	3.62	21.78	0.80

**Table 2:** Setting of parameters used for developing models.

**Models**	**Parameters**	**Range**
SVR	*C* and *g*	[0.25–128] and [0.25–16]
ANN	*N_h_* and *N_n_*	[1, 2] and [1–10]
RF	*N_t_* and *Minleafsize*	[1–100] and [1–10]
**Algorithms**	**Parameters**	**Range**
Ivy	Population sizes and iterations	[25, 50, 75, 100] and 200
BOIvy	Population sizes and iterations	[25, 50, 75, 100] and 200

**Table 3:** Iteration results of hybrid models optimized by the Ivy algorithm.

Models	Population Sizes			
	25	50	75	100
SVR	0.0562	0.0532	0.0553	0.0556
ANN	0.0600	0.0589	0.0595	0.0608
RF	0.0683	0.0669	0.0678	0.0670

**Table 4:** Iteration results of hybrid models optimized by the BOIvy algorithm.

Models	Population Sizes			
	25	50	75	100
SVR	0.0462	0.0467	0.0453	0.0476
ANN	0.0510	0.0488	0.0495	0.0515
RF	0.0643	0.0669	0.0638	0.0669

**Table 5:** Hyperparameters of hybrid models optimized by the Ivy algorithm.

Models	Optimal Hyperparameters	
	Ivy	BOIvy
SVR	*C*: 35.93; *g*: 0.57	*C*: 65.13; *g*: 0.96
ANN	*N_h_*: 2; *N_n_*: 4, 3	*N_h_*: 2; *N_n_*: 4, 5
RF	*N_t_*: 25; *Minleafsize*: 2	*N_t_*: 35; *Minleafsize*: 1

**Table 6:** Evaluation results for all models using the training set.

Models	Statistical Indices				
	R^2^	RMSE	U_1_	U_2_	VAF (%)
Ivy-SVR	0.9891	0.3305	0.0169	0.0046	98.9215
Ivy-ANN	0.9725	0.5238	0.0523	0.0108	97.3917
Ivy-RF	0.9482	0.7194	0.0738	0.0443	94.8176
BOIvy-SVR	0.9949	0.2267	0.0115	0.0021	99.4866
BOIvy-ANN	0.9895	0.4529	0.0456	0.0083	97.9553
BOIvy-RF	0.9713	0.5357	0.0544	0.0387	97.1284

**Table 7:** Evaluation results for all models using the test set.

Models	Statistical Indices				
	R^2^	RMSE	U_1_	U_2_	VAF (%)
Ivy-SVR	0.9682	0.8896	0.0684	0.0202	96.9198
Ivy-ANN	0.9231	1.3822	0.0947	0.0310	93.2350
Ivy-RF	0.8382	2.0055	0.3266	0.0683	84.1061
BOIvy-SVR	0.9756	0.7781	0.0582	0.0138	97.5657
BOIvy-ANN	0.9855	0.5998	0.0441	0.0077	98.5778
BOIvy-RF	0.8530	1.9115	0.3102	0.0373	85.5632

**Table 8:** Statistical results of errors calculated for each optimized model.

Models	Statistical Indices				
	Mean	St. D	Max	Min	Total
Ivy-SVR	0.4618	0.7718	3.8676	0.0031	15.7003
Ivy-ANN	0.7579	1.1733	4.7594	0.0038	25.7690
Ivy-RF	1.1281	1.6830	7.8208	0.0580	38.3588
BOIvy-SVR	0.4664	0.6321	2.8729	0.0047	15.8589
BOIvy-ANN	0.4313	0.4229	2.0187	0.0109	14.6676
BOIvy-RF	1.0947	1.5905	7.6847	0.0797	37.2226

## Data Availability

The original contributions presented in this study are included in the article. Further inquiries can be directed to the corresponding authors.
